# Daily physical activity and its contribution to the health-related quality of life of ambulatory individuals with chronic stroke

**DOI:** 10.1186/1477-7525-8-80

**Published:** 2010-08-03

**Authors:** Debbie Rand, Janice J Eng, Pei-Fang Tang, Chihya Hung, Jiann-Shing Jeng

**Affiliations:** 1 Department of Physical Therapy, University of British Columbia & Rehab Research Lab, GF Strong Rehab Centre, Vancouver, Canada; 2 School and Graduate Institute of Physical Therapy, National Taiwan University, and Physical Therapy Center and Department of Physical Medicine and Rehabilitation, National Taiwan University Hospital and National Taiwan University College of Medicine, Taipei, Taiwan ROC; 3 Department of Neurology, National Taiwan University Hospital and National Taiwan University College of Medicine, Taipei, Taiwan ROC; 4 International Collaboration on Repair Discoveries, Vancouver, Canada

## Abstract

**Background:**

Participation in daily physical activity (PA) post-stroke has not previously been investigated as a possible explanatory variable of health-related quality of life (HRQL). The aims were 1) to determine the contribution of daily PA to the HRQL of individuals with chronic stroke and 2) to assess the relationship between the functional ability of these individuals to the amount of daily PA.

**Methods:**

The amount of daily PA of forty adults with chronic stroke (mean age 66.5 ± 9.6 years) was monitored using two measures. Accelerometers (Actical) were worn on the hip for three consecutive days in conjunction with a self-report questionnaire [the PA Scale for Individuals with Physical Disabilities (PASIPD)]. The daily physical activity was measured as the mean total accelerometer activity counts/day and the PASIPD scores as the metabolic equivalent (MET) hr/day. HRQL was assessed by the Physical and Mental composite scores of the Medical Outcomes Study Short-Form 36 (SF-36) in addition to the functional ability of the participants. Correlation and regression analyses were performed.

**Results:**

After controlling for the severity of the motor impairment, the amount of daily PA, as assessed by the PASIPD and accelerometers, was found to independently contribute to 10-12% of the variance of the Physical Composite Score of the SF-36. No significant relationship was found between PA and the Mental Composite Score of the SF-36.The functional ability of the participants was found to be correlated to the amount of daily PA (r = 0.33 - 0.67, p < 0.01).

**Conclusion:**

The results suggest that daily PA is associated with better HRQL (as assessed by the Physical composite score of the SF-36) for people living with stroke. Daily PA should be encouraged to potentially increase HRQL. Accelerometers in conjunction with a self-report questionnaire may provide important measures of PA which can be monitored and modified, and potentially influence HRQL.

## Background

Health related quality of life (HRQL) is a multidimensional measure to quantify the burden of a disease from the point of view of the person with a disability[1,2]. Measures of physical function such as improved motor function, balance function, gait and independence in performing basic and instrumental activities of daily living have been recently reported to correlate significantly to better HRQL of individuals with chronic stroke[3]. However, it is not known whether daily physical activity (PA) is associated with higher HRQL in individuals with stroke.

Regular PA can prevent the development of secondary conditions such as obesity, depression, fractures, osteoarthritis, and osteoporosis[4], reduce morbidity and prevent recurrent stroke[5]. Since approximately 30% of individuals with stroke are at risk of sustaining a second stroke[6], PA for this population is of paramount importance[7,8]. Despite this fact, only a few studies have measured the amount of PA of individuals with stroke[9-13]. Few older adults with stroke achieve the recommended PA level of 1,000 kcal per week[9] and they undertake much lower levels of PA compared to healthy individuals, possibly due to their motor impairment[10-13].

Healthy older adults who report participation in regular PA of moderate intensity have been reported to have higher HRQL compared to healthy adults who were less physically active[14]. In addition, engaging in PA (assessed by self-report) has been found to positively impact the HRQL of older individuals with chronic conditions[9] and arthritis[15] and result in more healthy days for individuals with stroke[16].

The level of PA is a potentially modifiable factor (which can be changed, as opposed to age, for example), and yet the relationship of this variable to HRQL in individuals with stroke is unknown. Thus, the aims of our study were 1) to determine the contribution of daily PA to the HRQL of individuals with chronic stroke living in the community and 2) to assess the relationship between the functional ability (motor impairments of lower extremity, balance and walking distance) of these individuals to the amount of daily PA they undertake. This will enhance our understanding and identify the level of functional ability of individuals that really enables increased daily PA.

## Methods

This data has been used previously to establish the reliability of the accelerometers with individuals with chronic stroke [17]. The current study focused on a different research question. Study procedures were approved by local university and hospital research ethics boards and all eligible subjects gave written informed consent prior to participating in the study.

### Population

Forty adults with stroke (13 women and 27 men) volunteered to participate in the study. Inclusion criteria included: at least 6 months post stroke, living in the community, being able to walk independently (with or without a walking aid) and intact cognition [Mini Mental State Examination (MMSE)[18] score above 24 points]. Participants were excluded if they had a neurological condition other than stroke, major musculoskeletal condition (e.g., rheumatoid arthritis) or were not independent in basic activities of daily life (such as dressing or walking) before their stroke. Participants with a diagnosis of stroke were recruited from the local hospital database where they had previously received in-patient stroke rehabilitation. Fifty people were willing to volunteer for the study. Of these, 5 subjects dropped out prior to the data collection, 3 were excluded because their MMSE was less than 25 points and 2 subjects were eliminated upon checking the integrity of the accelerometer data (e.g., no activity recorded and perhaps were not wearing the device).

### Instruments and Study procedure

HRQL was assessed using the Medical Outcomes Study Short-Form 36 (SF-36)[19]. This is a self-report questionnaire containing 36 items that yield two summary scores- the Physical and Mental Composite Scores. The Physical Composite Score comprises 4 domains (physical functioning, role limitations due to physical problems, bodily pain, and general health). The Mental Composite Score comprises vitality, social functioning, role-emotion and mental health. Higher scores indicate a higher perceived health-related quality of life. The SF-36 has been found to have satisfactory reliability and validity in individuals with stroke[20].

PA was measured by triaxial accelerometers[21] to obtain a real-time measure in addition to a self report questionnaire. Actical accelerometer (Actical™ , MM; Mini-Mitter Co.) is a small (28 × 27 × 10 mm), waterproof sensor, which weighs only 17 g and can detect human movement (frequency range of 0.3-3 Hz, sensitive to 0.05-2.0 G-force, samples at 32 Hz). It detects acceleration in all 3 planes (although it is more sensitive in the vertical direction). Data were rectified, integrated and stored as activity counts every 15 seconds. Actical accelerometers have been found to have higher intra-instrument and inter-instrument reliability compared to the other two commonly used accelerometers (Actigraph and RT3)[22]. It has also been found to have excellent test-retest reliability (ICC > 0.95) over three days when worn at home by 40 participants with stroke[17] and during vigorous activities (ICC = 0.75-0.90) with individuals with Multiple Sclerosis (MS)[23,24]. Participants were given two accelerometers (one for each hip) attached to a hip belt positioned over the Anterior Superior Iliac Spine and were instructed to wear them for the waking hours of three consecutive days starting from the following day (the activity between week days and weekend days was not significantly different). The total activity kilocounts per day over 3 consecutive days quantified the mean amount of hip movement (i.e. PA). Active energy expenditure (AEE) was also reported to allow comparison of our data to others as some studies report only EE (the mean AEE per day calculated from Actical regression equations using the accelerometer activity counts, subject's weight, height and age), Since no significant differences were found between the accelerometer readings on opposite hips[17], the data from the paretic hip were used for the analysis. On returning the accelerometers, subjects confirmed wearing the accelerometer for each of the three days and data were checked to ensure that activity patterns were appropriate. In addition they filled in the PA Scale for Individuals with Physical Disabilities (PASIPD) inquiring about their activities over the past 7 days.

The PASIPD[25] is a 13-item self-report questionnaire that captures PA in three domain areas (recreation, household, and occupational activities). The score for the PASIPD takes into consideration the average hours per day for each item multiplied by a metabolic equivalent (MET) value associated with the intensity of the activity. The scores range from 0.0 MET hr/day (not performing any activities) to 199.5 MET hr/day (performing all of the listed activities for the maximum amount of days and hours). The PASIPD has been found to be reliable and valid when used with individuals with disabilities (including 13 individuals with subacute and chronic stroke); test-retest reliability (r = 0.77, p < 0.05) and criterion validity when correlated to the Actigraph accelerometer (r = 0.30, p < 0.05) [26].

The functional ability of the individuals was determined using the following assessments. The lower extremity items of the Chedoke-McMaster Stroke Assessment (CMSA)[27] were used to determine the presence and severity of leg and foot motor impairments (maximum of 14 points with larger values indicating less motor impairment of the lower extremity). This assessment has good concurrent validity with the Fugl-Meyer Assessment of Sensorimotor Recovery[27] and moderate correlations with burden of care and activities of daily living [28]. The Berg Balance Scale (BBS)[29] was used to assess the ability of the participants to maintain balance while performing 14 functional tasks (maximum score of 56 points; higher scores indicating improved balance function). The BBS is a psychometrically sound measure for assessing balance in individuals poststroke with high test-retest (ICC = .98) and intrarater reliability (ICC = .97)[30].

The Six Minute Walk Test (6MWT)[31] was used to assess walking distance. For this test, individuals were requested to walk as far as possible during six minutes on a 30 meter long walking course. According to the assessment instructions, standard phrases of encouragement were provided once a minute when the examiner informed the individual how many minutes he/she had completed. If needed, individuals were allowed to slow down or sit to take a break but the stopwatch was not stopped. The number of meters walked within the six minutes was recorded; further distance walked indicated higher walking endurance. The 6MWT was found to have excellent test-retest reliability (ICC = 0.97) and has been found to be strongly correlated with gait speed (r = 0.89) and the locomotion section of the FIM (r = 0.69) of individuals undergoing rehabilitation [32] indicating its validity.

### Data Analysis

Descriptive statistics were used to describe the study population. The measures of PA and health-related quality of life variables were not normally distributed therefore the median and interquartile range (IQR) were presented and Spearman correlation coefficients were used to determine the strength of the associations between measures. Correlations ranging from 0.25 to 0.49 were considered fair and values of 0.5 to 0.75 were considered moderate to good relationships[33]. In order to determine the contribution of PA (independent variable) to the Physical Composite Score of HRQL (dependent variable), we first controlled for the level of the motor impairment of the lower extremity as one measure of stroke severity, since this may impact the amount of daily PA. Next, we entered in the amount of daily PA using the accelerometer reading. For the second multiple regression model, we entered in the amount of PA using the PASIPD, after controlling for motor impairment. The dependent variable for the third and fourth regression models was the Mental Composite Score of the HRQL. The data were analyzed using SPSS (Windows version 15.0).

## Results

The mean age of the forty participants (13 men and 27 women) was 66.5 ± 9.6 years (range 49-82 years). They were 2.9 ± 2.4 years after stroke onset, with an equal division of left and right cerebrovascular accident. The mean (SD) Body Mass Index (BMI) (BMI = kg/m^2^) of the subjects was in the normal range (24.3 ± 3.6). They all had intact cognitive abilities based on the MMSE (27 ± 3 points, range 24-30 points). All of the participants could walk independently; 12 used a walking cane. Most of the participants had a near maximum score on the CMSA and BBS (Table 1) and thus a mild motor impairment. Despite this, a large variation in the amount of daily PA was seen. The median (IQR) total kilocounts per day was 21.5 (10.4-74.9) kilocounts/day. According to the PASIPD, the level of PA was low, median (IQR) 10.3 (6.1-17.1) MET hr/day out of the maximum possible 199.5 MET hr/day. The MET for the leisure activities (walking, exercising, participating in light/moderate/strenuous sports) is higher compared to household activities and work (Table 1). Only 5 participants reported they engaged in work or volunteer related activities. A fair significant correlation between the accelerometer activity kilocounts and AEE to the PASIPD was found (r = 0.45, p < 0.01 and r = 0.46, p < 0.01 respectively).

**Table 1:** The median and interquartile range (IQR) of the functional ability and PA measures

	Variable	Median	IQR
	6MWT (distance in meters)	345.5	264.0-418.7
	
**Functional ability measures**	Berg balance Scale (max 56 points)	54.0	50.2-56.0
	
	Chedoke-McMaster leg and foot impairment (max 14 points)	14.0	14.0-14.0

	Accelerometer - Total activity kilocounts/day	21.5	10.4-74.9
	Active Energy expenditure (kcal/day)	98.1	60.8-245.7
	
	PASIPD (MET hr/d) (max 199.5)	10.3	6.1-17.1
**PA Measures**	PASIPD Categories (items) (min-max possible MET hr/d)		
	Leisure Activities (1-6) (0 - 98.6)	4.5	2.4-10.9
	Household activities (7-12) (0 - 81.5)	0.6	0.0-2.3
	Work/Volunteer (13) (0 - 19.2)	0.0	0.0-0.0

6MWT - 6-minute walk test; Chedoke-McMaster leg and foot impairment- max 14 points = no lower extremity motor impairment

HRQL as assessed by the SF-36 was 39.4 points (33.3-53.9) for the Physical Composite Score and 43.4 points (64.2-50.3) for the Mental Composite Score. These scores are below the norm when compared to the median scores of healthy population (42.6 and 55.7 respectively)[18].

The participant's functional ability was found to be significantly correlated to PA (r = 0.45-0.67, p < 0.01) as measured by the hip accelerometers (Table 2). However, balance function was the only component of functional ability that was significantly correlated (r = 0.33, p < 0.05) to PA as measured by the PASIPD.

**Table 2:** Spearman correlations of the amount of daily PA with HRQL and functional ability

		PHYSICAL ACTIVITY			
		
		PASIPD		Accelerometer Activity kilocounts	
		
		r	p	r	P
**HRQL**	SF-36 Physical Composite Score	0.33	0.037	0.42	0.008
	
	SF-36 Mental Composite Score	0.03	0.84	0.05	0.7

	Chedoke lower extremity impairment	0.26	0.102	0.45	0.003
	
**Functional ability**	Berg Balance Scale	0.33	0.033	0.53	0.001
	
	6MWT (distance)	0.31	0.057	0.67	0.000

**Figure 1:**
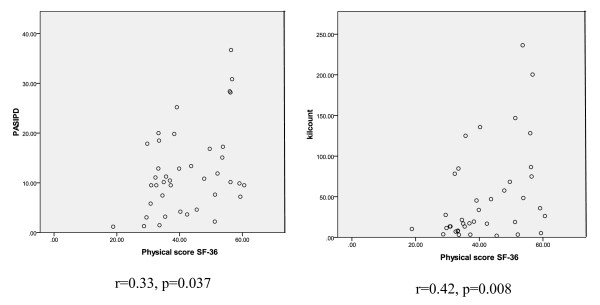
**Scatter data plots of the correlations between PA as assessed by the PASIPD (left) and the Accelerometer (right) to the Physical Composite Score of the SF-36**.

PA, as assessed by the accelerometer (r = 0.43, p < 0.01) and the PASIPD (r = 0.33, p < 0.05), was also found to be significantly correlated to the Physical Composite Score (Figure 1), but not the Mental Composite Score of the SF-36 (Table 2). Due to this fact, linear regression models for the Mental Composite Score of the SF-36 were not carried out. In addition, age and gender of the participants did not correlate to the Physical or Mental Composite Scores of the SF-36 and were not entered into the regression models. Using linear regression, lower extremity impairment was first entered to control for the stroke motor severity and found to account for 13% (p = 0.02) of the total variance of the Physical Composite Score of the SF-36. Adding PA as assessed by the PASIPD resulted in an R^2 ^change of 12% (p = 0.017). The total variance accounted by the final model was 26% (Table 3). In the second model, adding PA as assessed by the accelerometer activity counts after controlling for motor impairment resulted in an R^2 ^change of 10% and significantly improved the model (p = 0.034). The total variance accounted by the final model was 23.4% (Table 3).

**Table 3:** Linear regression models for determining the contribution of PA on the physical composite score of the SF-36 after controlling for lower extremity impairment

		**R**^**2**^	**R**^**2 **^**change**	Unstandardized ß (standard error)	Standardized ß	P
**Model 1**	Lower extremity impairment	0.134	0.134	1.53 (.632)	0.367	0.020
	
	Lower extremity impairment	0.134	0.134	1.33 (.598)	0.367	0.020
	PASIPD	0.260	0.126	0.457 (.182)	0.358	0.017

	Lower extremity impairment	0.134	0.134	1.53 (.632)	0.367	0.020
	
**Model 2**	Lower extremity impairment	0.130	0.130	1.2 (.626)	0.301	0.024
	Accelerometer activity kilocounts	0.234	0.104	6.41 (0.00)	0.337	0.034

## Discussion

Accelerometers in conjunction with a self-report questionnaire were used to assess the daily PA of 40 ambulatory individuals with chronic stroke living in the community. Daily PA (after controlling for lower extremity impairment) explained 10-12% of the variance of the physical (but not the mental) composite score of the SF-36. Overall low levels of daily PA were revealed for these individuals with a mild motor impairment.

The median AEE from the hip accelerometer of our participants was 98 kcal/day, which is lower than the EE reported by Haeuber (2004)[14] of 17 individuals with chronic stroke of similar age (321 ± 187 kcal/day). The range of the AEE is vast reflecting that some subjects likely spent most of their days sitting in a chair (20 kilocounts/day) while others were relatively active (236.8 kilocounts/day). According to the US Surgeon General's 1996 report, approximately 1,000 kilocalories/week (150 kilocalories/day) is associated with substantial health benefits and this activity does not need to be vigorous to achieve benefit[34]. Sixty percent of our cohort of individuals with mild motor impairment did not meet this recommended level of PA. The lack of PA in community dwelling people with stroke has been reported previously[10,13,22].

The median activity level of our cohort as measured with the accelerometer is 21.5 kilocounts/day. For comparison, the median (IQR) activity level as measured with Actical accelerometers of 40 older adults (mean age 71.3 ± 3.8 years) living in the community who walked a median 5202 steps/day, was 377.3 (236.5-502.2) kilocounts/day (our unpublished data), which is more than 15 times more than the individuals with stroke.

The level of PA as assessed by the PASIPD was also found to be low for our cohort (10.3 METs hr/day) although comparable to the findings of the PASIPD of 209 older adults with multiple chronic conditions (11.0 ± 7.8 METs hr/day)[35] and 45 individuals with neurologic and orthopedic conditions (15.5 ± 10.6 METs hr/day)[26].

The health-related quality of life of individuals is known to be influenced by a stroke[36]. Our findings support previous literature since the median scores the mean Physical and Mental Composite scores of the SF-36 of our community-dwelling sample with mild motor impairment were lower than the norms. The scores of the SF-36 are also comparable to scores of individuals with mild stroke (N = 14) 3 months post-stroke but higher than individuals with moderate stroke (N = 15)[19].

Daily PA of our cohort explained 23-26% in the variance of the individual's HRQL after controlling for lower extremity impairment. Improved HRQL is expected to be supplementary to the other well known health benefits of PA[5] and our results emphasize the importance of PA after stroke. PA has been reported to improve motor function, ADL and decrease the symptoms of depression, which possibly results in an increase in the HRQL. It is possible that the reported physical activities were undertaken in the community with others and this social interaction may influence HRQL. However a large amount of variance in HRQL remains unexplained. Factors such as cognitive performance, mood, social support and socioeconomic status which were not addressed in this study, may contribute to the HRQL as well.

The self report measure (PASIPD) explained similar variance in HRQL as the objective measure of the accelerometer. This may be due to the fact that our subjects were not physically active and therefore their self report was relatively accurate. In addition it is possible that one's HRQL is based mainly on one's perception of the activities he or she engages in such as sport and leisure activities (captured by the PASIPD) and not basic activities such as dressing and walking around the house (captured only by the accelerometer). A previous study reported lower levels of activity obtained by real-time accelerometers compared to higher self-report recall from 1114 healthy adults (ages 18-69)[37]. Our correlation (r = 0.45) is comparable to that reported previously between the PASIPD and Actigraph accelerometer in individuals with neurologic and orthopedic conditions[24]. Due to the unique attributes of each measure, it may be useful in future studies to use both measures to capture accurate levels of PA[38,26].

Improving quality of life is the most important goal of rehabilitation and community re-integration after a stroke. To our knowledge, this is the first study to report the independent contribution of daily PA measured by accelerometers and a self report questionnaire to the HRQL of individuals with stroke. These findings are in accordance with the findings from healthy older individuals and also support the findings of Sawatzy et al.[10] which found that more self-report leisure-time PA reduced the negative impact of stroke on the mobility component of the Health Utility Index (HUI), but not the emotional well being component of the HUI. Since we revealed a positive relationship between PA and the Physical Composite Score, individuals with mild motor impairment should be encouraged to be more physically active including increasing walking activities, as one avenue of enhancing their HRQL. Counseling these individuals to participate in PA[13,26,39] or in exercise programs[40] is important. Recent studies have also used pedometers as a feedback tool to increase walking in healthy individuals[41,42] and sedentary adults[43]. While some of the factors reported to contribute to HRQL are not modifiable (e.g. age), other factors are more difficult to modify after stroke (e.g. severity of neurological impairment) and some factors are often difficult to improve, especially at the chronic stage (e.g. functional ability). Therefore in order to increase the HRQL, it might be feasible to increase the daily PA, especially in ambulatory individuals. Further follow-up studies are needed to determine if an increase in the level of daily PA (and not only improved functional ability) will in fact generate an increase in HRQL.

All of the functional ability measures were found to correlate to the amount of PA, indicating that greater balance function and decreased motor impairment can enable daily PA, leisure and recreation activities. The strongest association between PA as assessed by the accelerometers was found with the distance walked in the 6MWT. In contrast, the amount of PA according to the PASIPD was significantly correlated only to balance function. This may be due to the fact that the almost half of the PASIPD items includes activities such as household tasks that may not substantially involve walking or lower extremity function, but do require balance function (e.g. washing dishes).

### Limitations of the study

As our study is cross-sectional, it is not possible to determine causation between PA and HRQL. The results of this study can be generalized only to individuals who regain their walking ability post stroke, which is approximately 70% of all individuals post stroke[44]. A limitation of the accelerometers is that the type of movements performed by the subject is not known. Thus, we cannot distinguish between walking versus another activity such as moving within a chair. However, all such movements will contribute to PA that is beneficial for health.

## Conclusions

daily PA (measured by an accelerometer and self-report questionnaire) contributes to better HRQL for people living with stroke (as assessed by the Physical composite score of the SF-36. In addition, functional ability is associated with the amount of participation of PA.

## Competing interests

The authors declare that they have no competing interests.

## Authors' contributions

JJE and PT conceived the study, CH, PT, JJE, JJ participated in data collection of the study, DR conducted data analysis, DR and JJE participated in interpretation of data and manuscript preparation. All of the authors reviewed the manuscript prior to submission.
